# Post-Release Deformation and Motion Control of Photonic Waveguide Beams by Tuneable Electrothermal Actuators in Thick SiO_2_

**DOI:** 10.3390/mi9100496

**Published:** 2018-09-27

**Authors:** Kai Wu, Marcel Tichem

**Affiliations:** Precision and Microsystems Engineering (PME), Delft University of Technology, Mekelweg 2, 2628 CD Delft, The Netherlands; kai.wu@tudelft.nl

**Keywords:** photonic packaging, flip-chip assembly, silicon dioxide, polycrystalline silicon, MEMS, bimorph actuator, electrothermal actuator, multilayer beam, post-release deformation, out-of-plane motion, photonic waveguide alignment

## Abstract

Photonic packaging, which includes high-precision assembly of photonic sub-systems, is currently a bottleneck in the development of commercially-available integrated photonic products. In the pursuit of a fully-automated, high-precision, and cost-effective photonic alignment scheme for two multi-channel photonic chips, this paper explores different designs of the on-chip electrothermal actuators for positioning mechanically-flexible waveguide structures. The final alignment goal is ∼100 nm waveguide to waveguide. The on-chip actuators, particularly for out-of-plane actuation, are built in a 16 μm-thick SiO${}_{2}$ photonic-material stack with 5 μm-thick poly-Si as an electrothermal element. A major challenge of out-of-plane positioning is a 6 μm height difference of the waveguides to be aligned, due to different built-up material stacks, together with a misalignment tolerance of 1 μm–2 μm from the pre-assembly (flip-chip) process. Therefore, the bimorph-actuator design needs to compensate this height difference, and provide sufficient motion to align the waveguides. We propose to exploit the post-release deformation of so-called short-loop bimorph actuator designs to meet these joint demands. We explore different design variants based on the heater location and the integration of actuator beams with waveguide beams. The actuator design (with 30 μm poly-Si and 900 μm SiO${}_{2}$ in length) has ∼8 μm out-of-plane deflection and is able to generate ∼4 μm motion, which meets the design goal.

## 1. Introduction

Photonic integration technology, i.e., the design and microfabrication of on-chip optical functions, is key to establishing advanced applications in, e.g., data communication and sensing. Several material platforms are available to create Photonic Integrated Circuits (PICs), including indium phosphide (InP) [1] and silicon dioxide/silicon nitride (SiO${}_{2}$/Si${}_{3}$N${}_{4}$) [2]. Today, PICs with complex functionality can be designed and fabricated to contain both passive (waveguides, splitters) and active (lasers, detectors) optical functions.

Generic foundry-based processes in the photonic domain have brought PIC costs within the scope of many applications, i.e., ∼€10–100 per chip [1,3,4]. A key bottleneck for market entrance is volume-compatible integration, i.e., assembling one or more PICs together with other optical and electrical components into a single housing [5]. Packaging requires the establishment of opto-mechanical connections, electronic interconnections and thermal management. The standardization of packaging and the automation of assembly processes for photonic products are still in their infancy. The current photonic packaging and assembly technologies are mainly based on custom-engineered solutions, which makes device integration an order of magnitude more expensive than the PIC cost [3]. Particularly, the fine-alignment of optical components to maximize light coupling is a major challenge.

**Figure 1:**
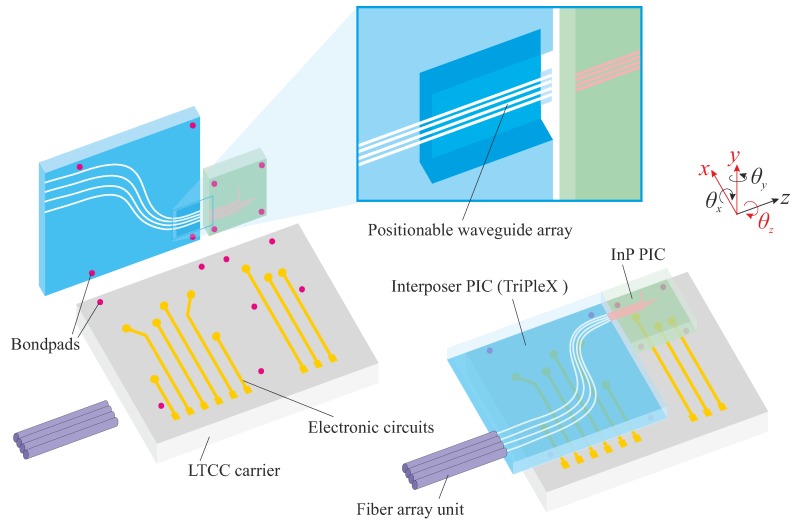
Overview of two-photonic integrated circuit (PIC) assembly: the TriPleX and InP chips are flip-chip bonded on top of the Low Temperature Co-fired Ceramics (LTCC) carrier, attaching with a fiber array unit. After this, the mechanically-flexible waveguide beams are fine-aligned by on-chip MEMS.

**Figure 2:**
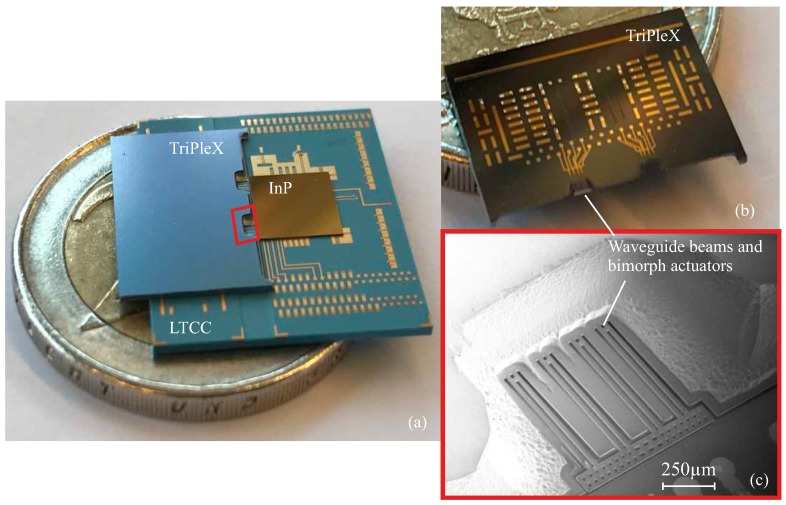
(**a**) photograph of two PICs flip-chip assembled on the LTCC carrier, with a 2-euro coin as a reference; (**b**) photograph of the top view of the TriPleX PIC; (**c**) SEM (scanning electron microscope) image of the waveguides and bimorph actuators from the bottom view of the TriPleX PIC.

The PHASTFlex project (Photonic Hybrid ASsembly Through Flexible Waveguides) aims for a novel photonic alignment scheme for multi-port PICs. The goal is to provide a fully-automated, high-precision and cost-effective assembly approach for next generation multi-PIC hybrid photonic packages [3,6]. The alignment involves two steps (Figure 1). The first step is a flip-chip bonding process, involving automatic pick and place of two PICs on a common carrier, with a-few-micrometer misalignment that can be compensated in a later step. One PIC is an InP-based PIC that contains active photonic functions. The other PIC is based on the TriPleX™(SiO${}_{2}$/Si${}_{3}$N${}_{4}$) platform with passive photonic materials [2]. The TriPleX PIC acts as an interposer between the InP PIC and a fiber array unit (FAU), and provides mode-size and waveguide-pitch conversion, and allows for integrating additional electronic functionality. A multilayer LTCC (Low Temperature Co-fired Ceramic) substrate is used as the common carrier, which provides high-speed RF (radio frequency)-signals and routing of large numbers of electrical I/O (input/output). In the second step, fine alignment of mechanically-flexible waveguide beams [7] is performed by chip-integrated MicroElectroMechanical Systems (MEMS). The aligned position is then maintained by MEMS locking functions. Figure 2 shows an assembly product after the first alignment step.

This paper focuses on the design and optimization of the MEMS functions, specifically for out-of-plane translation and rotation around the light-propagation axis (*y* and $\theta_{z}$ in Figure 1). Earlier work has proposed the use of electrothermal actuators [8,9,10], which are integrated with the ∼16 μm-thick (mechanically dominant) SiO${}_{2}$ TriPleX-material stack. Together with the SiO${}_{2}$ stack, the electrothermal actuators in this paper also include a 5 μm-thick layer of boron-doped polycrystalline silicon (poly-Si) that serves both as heater layer and structural layer.

**Figure 3:**
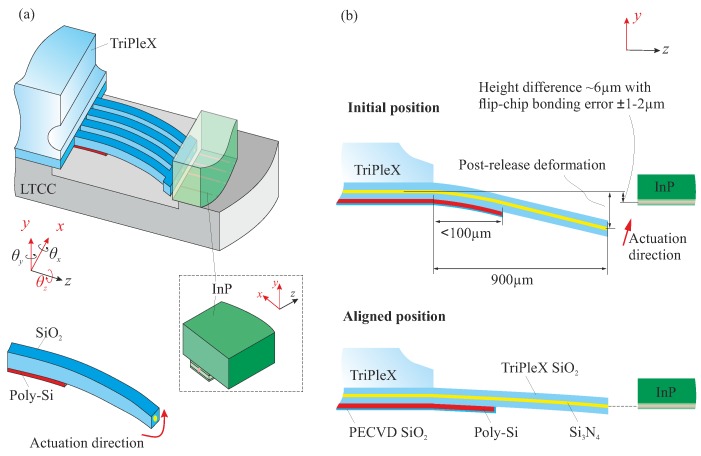
Illustration of the short-loop bimorph actuator as a means to compensate the height difference of the waveguide end facets and to provide a sufficient motion range (bondpads are absent). (**a**) close-up of the interface among the PICs and LTCC carrier; (**b**) 2D view of the initial position of two waveguides after the flip-chip assembly, and the aligned position after the out-of-plane actuation.

A number of factors complicate the design of well-performing MEMS functions. First, extrinsic stress mainly from the thermal cycles in the fabrication, and intrinsic stress in the multilayer actuator structures, is significant. Thus, when the waveguide and actuator arrays are realized from a Si wafer, they deform significantly. In addition, when the TriPleX and InP PICs are bonded onto the LTCC carrier, a nominal offset between the waveguide cores in *y* direction of about 6 μm exists, due to the built-up material stacks, both in the optical layers and in the bondpads for flip-chip bonding (Figure 3). Thirdly, the precision (in *y* direction) of flip-chip assembling is in the order of ±1 μm–2 μm (chip to chip). All these factors complicate the design of the actuator system. The challenge is to obtain a final alignment precision of ∼100 nm, waveguide to waveguide. We demonstrate in this paper the intended use of the post-release deformation of actuator structures to tune the resulting initial position of the waveguide end-facets. At the same time, the actuators must meet the motion range demands.

Fabrication of movable waveguide structures in e.g., InP [11,12], polymer [13,14] and Si- [15] based platforms, has been demonstrated before. For the fine positioning in photonic packaging, MEMS are commonly based on silicon (Si), e.g., to control micro-lenses [16] and micro-mirrors [17]. Besides Si-MEMS, SiO${}_{2}$/aluminum (Al)-based electrothermal MEMS have been developed to carry out out-of-plane actuation [18,19]. An electrostatically-controlled thick SiO${}_{2}$-based waveguide system with in-plane motion has been reported before [20]. The SiO${}_{2}$-based MEMS design we propose has both in-plane and out-of-plane functions and a variable set of waveguide beams [21]. In the overall design, the MEMS structure occupies at least 1.5 mm${}^{2}$. We have developed novel and robust methods to realize such complex MEMS designs, and have developed design rules to cope with.

Earlier designs of the bimorph actuator, consisting of a poly-Si layer with almost the same length as the SiO${}_{2}$ beam, have shown more than 60 μm post-release deflection, while the achieved motion within the safe operating range was measured to be less than 20 μm [8,9,10]. A short-section poly-Si bimorph actuator, i.e., an actuator where the poly-Si track is shorter than the SiO${}_{2}$ beam, was suggested before [22]. The initial results suggested that the poly-Si length is a key parameter to impact both the post-release deformation and the motion range of the actuator. In this paper, we aim to systematically understand and quantify the contribution of the poly-Si length to both post-release deformation and motion of the bimorph actuator. Additionally, we relate the experimental results to the analytical and numerical models.

Firstly, the proposed short-loop bimorph actuators with the main design variants are introduced in Section 2. Then, Section 3 shows the experimental characterization work, together with analytical simulation. In Section 4, the results are discussed and Section 5 provides conclusions.

## 2. Design and Fabrication

### 2.1. Design Variants

**Figure 4:**
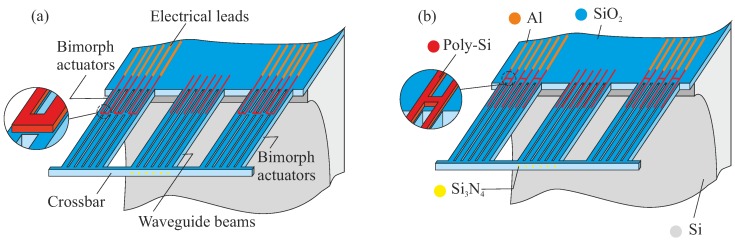
Conceptual representation of an array of waveguide (WG) beams (50 μm pitch), positioned by two different designs of bimorph actuators (see the zoom-in parts): (**a**) bridge design with configuration of separate bimorph actuator and (**b**) non-bridge design with configuration of separate bimorph actuator.

**Figure 5:**
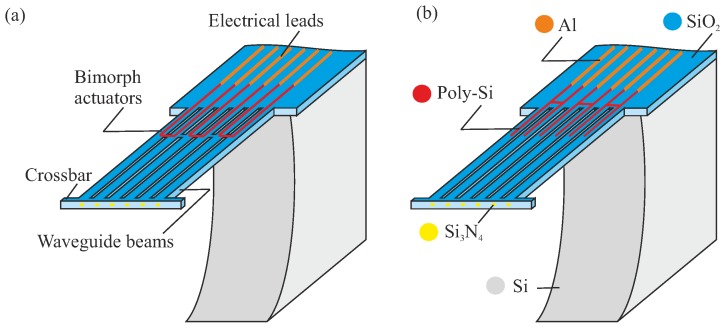
Conceptual representation of an array of waveguide (WG) beams (50 μm pitch), positioned by two different designs of bimorph actuators: (**a**) bridge design with configuration of integrated bimorph actuator and (**b**) non-bridge design with configuration of integrated bimorph actuator.

The core of the design consists of a set of waveguide (WG) beams and actuator beams. As far as the SiO${}_{2}$ stack is concerned, these beams have the same dimensions (length, cross-section). The actuator beams are provided with a section of poly-Si (Figure 4 and Figure 5). They interact with the WG array through a crossbar which connects the beams at their free ends. This design preserves the lithographically-defined pitch between the WG end-facets, and supports the joint motion of all WG beams by a limited number of actuators. The mechanical coupling between actuator and WG beams obviously leads to cross-sensitivities during operation.

We explore two different designs of the poly-Si heater, based on its location. The poly-Si heater either forms a loop across two adjacent beams, or is placed on the chip body at the base of the suspended structure while straight poly-Si strips conduct the heat to the rest of the poly-Si track. These designs are referred to as bridge design (BD) and non-bridge design (NBD), respectively. Their motion performance will be different. The heaters can be powered until a maximum allowable temperature is reached. In the NBD (Figure 4b and Figure 5b), some of the heat will be conducted into the Si chip, and will therefore not effectively contribute to the bimorph’s motion. The BD variant (Figure 4a and Figure 5a) heats up more effectively along the entire heater structure. Modeling and experimental results presented in later sections confirm this expectation.

Another design variation concerns the integration of actuators in the overall design. The actuator beams are either placed adjacent to the WG beam array, or are integrated with the WG beams. These configurations are referred to as separate bimorph actuator (SBA) configuration or integrated bimorph actuator (IBA) configuration, respectively. Figure 4 presents both BD and NBD with the SBA configuration, which has a (mechanically) more compliant crossbar. As a result, this can allow more degree of rotational adjustment. Figure 5 shows both BD and NBD with the IBA configuration. In Appendix A, Table A1 summarizes the main design parameters and values for the MEMS design.

### 2.2. Fabrication

For the purpose of this paper, it is relevant to understand the material layers which constitute the WG and actuator beams. The complete fabrication process flow is summarized in Appendix B.

Both the WG beams and the actuator beams are made of the TriPleX-material, which in itself is a stack of various SiO${}_{2}$ layers with a total thickness of about 16 μm. The WG beams contain a Si${}_{3}$N${}_{4}$ core. This core is only ∼200 nm in thickness, and plays no role of significance in the mechanical behavior of the beams.

To create an actuator structure, a 5 μm-thick poly-Si layer is deposited. The SiO${}_{2}$/poly-Si structures are patterned from the front side, and then are released from the bulk Si chip to form free-standing structures. During this release step, the poly-Si layer is protected by a 2 μm-thick ($t_{\mathrm{pox}}$) layer of SiO${}_{2}$ by plasma-enhanced chemical vapor deposition (PECVD). As the PECVD SiO${}_{2}$ stack causes additional post-release deflection, it is removed from the SiO${}_{2}$-only sections, i.e., beyond the poly-Si section of the beams.

**Figure 6:**
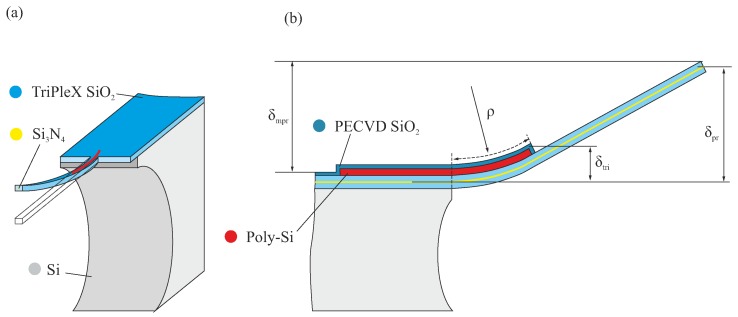
(**a**) schematic representation of a curved multilayer beam; (**b**) 2D illustration of the definition of the post-release deformation.

Hence, the structural stacks which determine the post-release deformation of the beams essentially consist of 16 μm-thick SiO${}_{2}$, 5 μm-thick poly-Si, and 2 μm-thick PECVD SiO${}_{2}$ (Figure 6).

### 2.3. Basic Models

Estimation of the vertical post-release-position of a beam’s end-facet is done in two steps. First, the curvature of the trilayer section is computed using a multilayer-beam model [23,24]. The model describes the curvature of a multilayer beam depending on dimensions and material properties (Young’s modulus, coefficient of thermal expansion) of the layers, as well as strains that result both from thermal and residual/intrinsic stress. The thermal stress results from a considered temperature difference (ΔT). To find the post-release deformation, the difference between the deposition temperature of each layer and the ambient temperature needs to be considered. For estimating the motion range, the difference between the ambient temperature and the operation temperature needs to be used. Details of the model can be found in Appendix C. The computed curvature applies to the trilayer section of the beams. To compute the end-facet position of this section, the following equation is used:

(1) $$\delta_{\mathrm{tri}}=\rho \left(1-\cos\left(\frac{L_{\mathrm{poly}}}{\rho }\right)\right),$$

where $L_{\mathrm{poly}}$ is the length of poly-Si on the suspended beam structure, and ρ is the radius of curvature of the trilayer stacks (Figure 6).

Second, the remaining SiO${}_{2}$ stack is considered straight by approximation; the small curvature in the SiO${}_{2}$-only sections resulting from the intrinsic stress gradient can be ignored. Hence, the end-position of the entire beam can be approximated by

(2) $$\delta_{\mathrm{pr}}=\delta_{\mathrm{tri}}+\left(L_{\mathrm{wg}}-L_{\mathrm{poly}}\right)\sin\left(\frac{L_{\mathrm{poly}}}{\rho }\right),$$

where $L_{\mathrm{wg}}$ is the length of the entire suspended cantilever.

**Figure 7:**
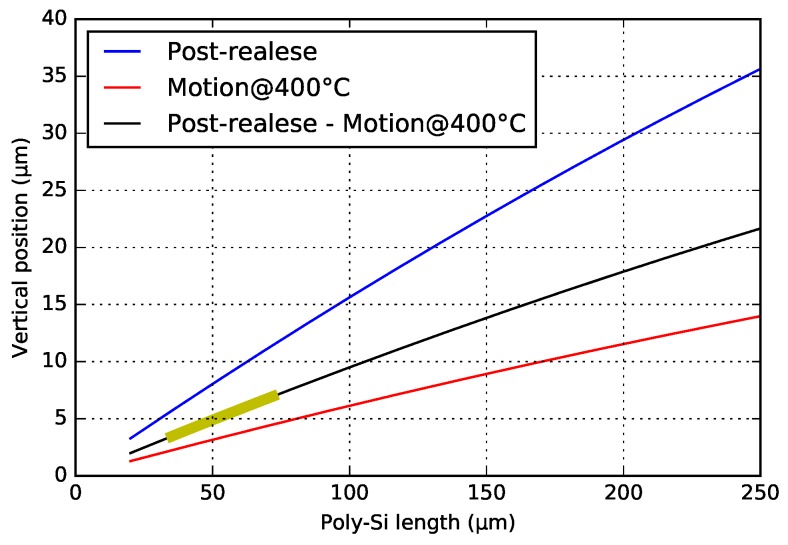
Analytical simulation (excluding intrinsic stress) of post-release deformation and motion. The bold-yellow line indicates the desired post-release end-positions (4 μm–8 μm) of the beam model, and the corresponding poly-Si lengths of 30 μm–75 μm.

If only considering the thermal effects from the fabrication, there are two major temperature cycles: the epitaxial poly-Si growth at 1050 ${}^{\circ }C$ and later the PECVD SiO${}_{2}$ deposition at 400 ${}^{\circ }C$. By substituting these temperature values into the formulas in Appendix C, the post-release deformation can be computed. For simulating the actuation, the same model can be used as well, and a possible poly-Si operation temperature (e.g., 400 ${}^{\circ }C$, which is far below the poly-Si recrystallization temperature ∼600 ${}^{\circ }C$ [25,26]) is considered. Figure 7 shows the simulation results for post-release deformation and motion, and suggests that $L_{\mathrm{poly}}$ of 30 μm–75 μm, with a total SiO${}_{2}$ beam length of 900 μm, can achieve the design goal. The total beam length of 900 μm was chosen, since this gave a good fabrication yield and low stiffness for the entire WG beam and actuator array.

## 3. Experimental Results

This section summarizes the characterization of the various bimorph actuator designs. First, the post-release deformation and motion range of actuator beam arrays are characterized for varying $L_{\mathrm{poly}}$ on post-release deformation (Section 3.1). Then, the motion behavior of the bridge design (BD) and non-bridge design (NBD) actuator is compared (Section 3.2). Moreover, the integrated bimorph actuator (IBA) and separate bimorph actuator (SBA) designs are compared, focusing on their post-release deformation (Section 3.3) and rotational actuation (Section 3.4).

The post-release deformation was characterized by a white-light interferometer, Contour GT-K 3D optical profilometer (Bruker Corporation, Billerica, MA, USA) with ∼10 nm–50 nm resolution. The motion range was measured using a Polytec MSA-400 vibrometer (Polytec GmbH, Waldbronn, Germany) with ∼50 nm–100 nm resolution in low frequencies. The Keithley 2611 source meter (Keithley Instruments, Inc., Cleveland, OH, USA) was used to drive the actuators.

### 3.1. Varying Poly-Si Length versus Post-Release Deformation

The post-release deformation ($\delta_{\mathrm{pr}}$) is obtained by measuring the position of the crossbar with respect to the top layer of the SiO${}_{2}$ stack. Since the PECVD oxide is locally removed from the beam, the thickness ($t_{\mathrm{pox}}$ = 2 μm) of that layer needs to be added. Thus, $\delta_{\mathrm{pr}}$ is the sum of $\delta_{\mathrm{mpr}}$ and the thickness of PECVD SiO${}_{2}$ (Figure 6):

(3) $$\delta_{\mathrm{pr}}=\delta_{m}\mathrm{pr}+t_{\mathrm{pox}},$$

where $\delta_{\mathrm{mpr}}$ is the distance from the top surface of the base to the top surface of the crossbar (free end).

**Figure 8:**
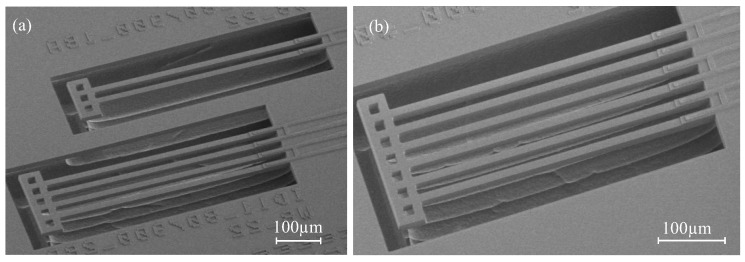
SEM images of non-bridge design (NBD) bimorph actuators with different pairs of actuator beams. (**a**) 1-pair and 2-pair bimorph actuators; (**b**) 3-pair bimorph actuators.

**Figure 9:**
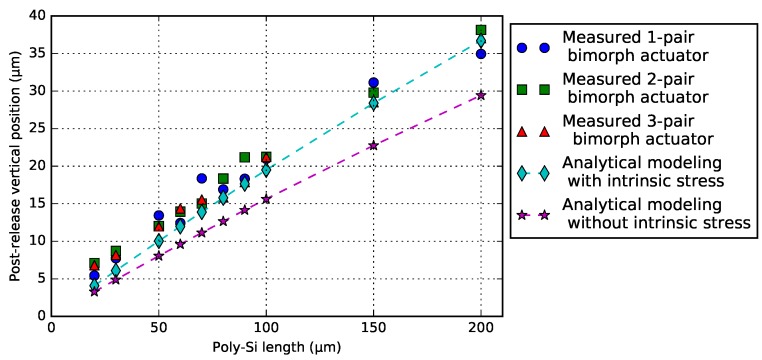
Experimental and analytical modeling results of post-release deformation of the NBD bimorph actuators.

The post-release deformation of a series of bimorph actuator designs, with various of poly-Si lengths, including 1-pair, 2-pair and 3-pair of NBD-actuator (50 μm pitch) variants (Figure 8), was measured. $L_{\mathrm{poly}}$ varies from 20 μm to 200 μm. The results are plotted in Figure 9. Each measurement point is the average result of three measurements on the free-end of the crossbar. A single-beam analytical model is included in the graph, both with and without considering intrinsic stress. Values for the intrinsic stress were estimated and summarized, and can be found in Table A2.

The analytical model that does consider the intrinsic stress is in a good agreement with the measurements. For a given $L_{\mathrm{poly}}$, the post-release deformation should be the same for each of the actuators. The minimum and maximum difference between all designs at the same value for $L_{\mathrm{poly}}$ is in the order of ∼1 μm–2 μm. As described in Section 1, for the specific PHASTFlex case, an offset of roughly 6 μm needs to be compensated. The results indicate that this is possible with a poly-Si range of 20 μm–50 μm.

### 3.2. Varying Poly-Si Length versus Motion

**Figure 10:**
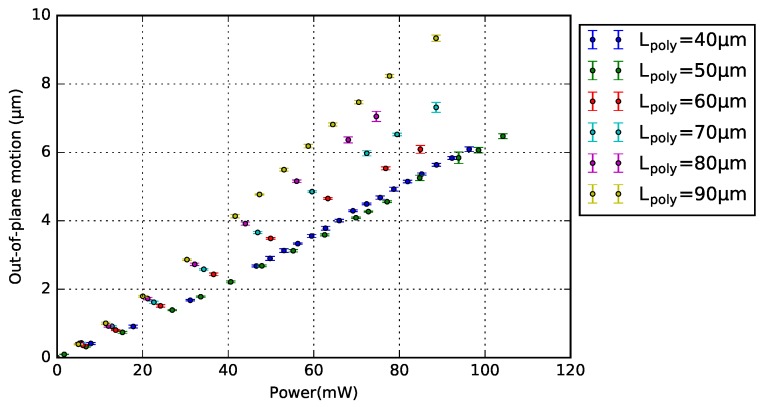
Motion characterization with standard deviation of the 1-pair bridge design (BD) bimorph actuator with different poly-Si lengths.

**Figure 11:**
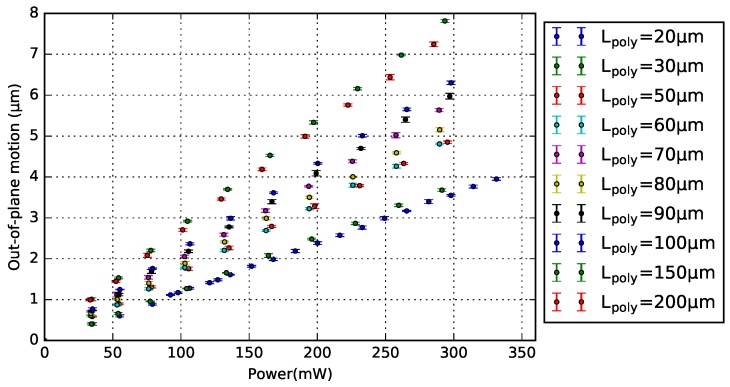
Motion characterization with standard deviation of the 1-pair NBD bimorph actuator with different poly-Si lengths.

The design of 1-pair bimorph actuator was chosen to analyze the relationship between $L_{\mathrm{poly}}$ and motion. Note that the number of actuator pairs does not influence the generated motion (Section 4.4). Figure 10 and Figure 11 show the motion measurements of a series of 1-pair BD and NBD bimorph actuators with varying $L_{\mathrm{poly}}$, respectively. Each measurement point is the average result of five measurements on the free-end of the crossbar, and their standard deviation is included. Both plots show the generated vertical motion as a linear function of its power consumption, and the motion increases with a longer $L_{\mathrm{poly}}$.

**Figure 12:**
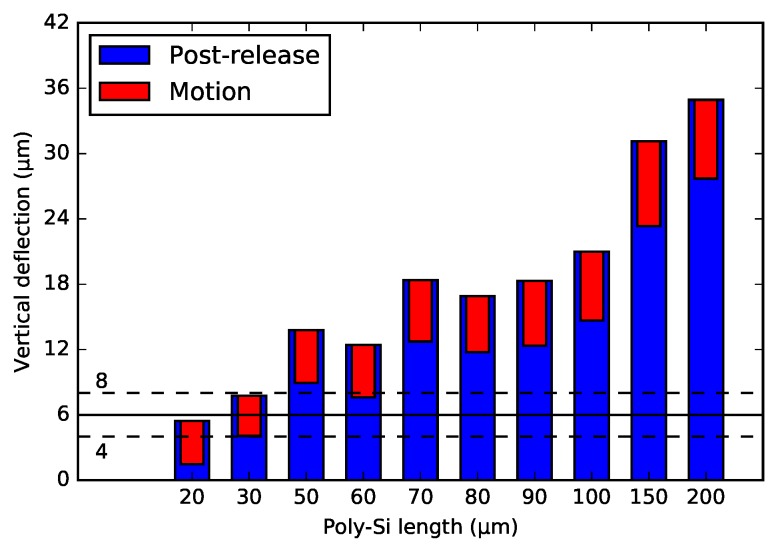
Summary of the end position of the 1-pair integrated bimorph actuators with the non-bridge design after the actuation. The solid line indicates the 6 μm nominal offset, with the maximum ±2 μm pre-assembly errors (dashed-line indication).

**Figure 13:**
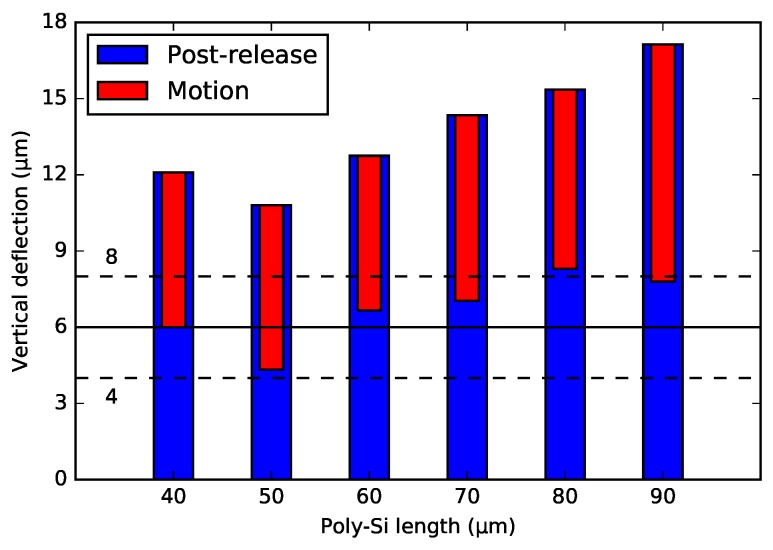
Summary of the end position of the 1-pair integrated bimorph actuators with the bridge design after the actuation. The solid line indicates the 6 μm nominal offset, with the maximum ±2 μm pre-assembly errors (dashed-line indication).

Figure 12 and Figure 13 show the post-release deflection and motion range for the NBD and the BD actuator, respectively. These graphs can be used to design WG arrays for selected specifications. The solid line points out the 6 μm nominal offset between the WG layers in the chip in the PHASTFlex case. The flip-chip assembly error in vertical direction is estimated to be ∼1 μm–2 μm (chip to chip). This implies that, in the worst case, the initial WG offset needs to be ∼8 μm (6 μm nominal WG layer offset plus 2 μm assembly error). The WG array needs to be designed for this situation, i.e., it needs to have an ∼8 μm initial offset. If the assembly error is in the other direction, the offset between the WG layers after assembly will be 4 μm (6 μm nominal WG layer offset minus 2 μm assembly error). Hence, the motion range must be such that this position is achieved, i.e., a motion range of 4 μm (8 μm targeted initial offset minus 4 μm) is needed. For these design specifications, designs with ∼30 μm poly-Si lengths are suitable.

There is a lower limit to the manufacturable poly-Si length, which is 40 μm for the BD variant. This is due to the need for perforations in the SiO${}_{2}$ structure for successful release that requires a perforation with a suitable dimension to realize the suspended beams. The perforation has the minimum dimension of ∼38 μm (determined by the dimension of the SiO${}_{2}$ beam). From this perspective, the NBD actuator shows more design freedom on choosing $L_{\mathrm{poly}}$.

### 3.3. Post-Release Deformation of Separate Bimorph Actuator (SBA) and Integrated Bimorph Actuator (IBA)

**Figure 14:**
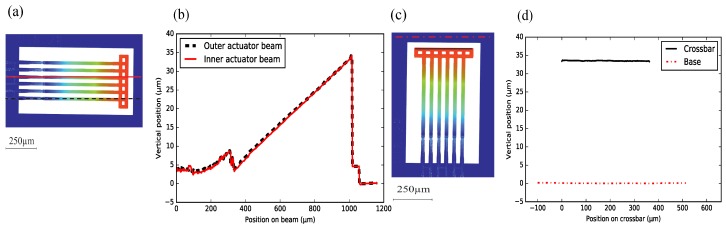
(**a**) top view of integrated bimorph actuator (IBA) with NBD (50 μm pitch), with the indication of the two measurement locations; (**b**) the post-release deflection measured over an inner and an outer actuator beams; (**c**) indication of two measurement locations; and (**d**) the height profiles of the crossbar and the base.

Figure 14 shows the height profile of an array of suspended beams of a NBD-IBA configuration ($W_{\mathrm{wgp}}$ = 50 μm, $L_{\mathrm{poly}}$ = 200 μm). The inner and outer WGs show the same post-release deflection, which is confirmed by the measured horizontally-flat crossbar, see Figure 14c,d). The post-release deflection of the beams is ∼35 μm, which matches the analytical result shown in Figure 9. The curvature of the poly-Si section was measured to be ∼230 /m, and the SiO${}_{2}$-only section remains almost straight (Figure 14a,b).

**Figure 15:**
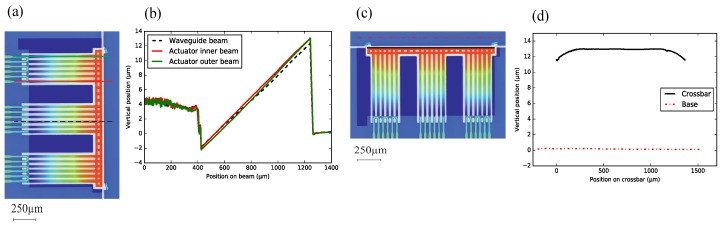
(**a**) top view of separate bimorph actuator (SBA) with bridge design (BD) (50 μm pitch), with the indication of the three measurement locations; (**b**) the post-release deflection of a WG and two actuator beams; (**c**) indication of two measurement locations; (**d**) the height profiles of the crossbar and the base.

For the SBA configuration ($W_{\mathrm{wgp}}$ = 50 μm, $L_{\mathrm{poly}}$ = 80 μm), with more suspended beams and a longer crossbar structure, the post-release deflection of inner beams (the inner WG and actuator beams) is still the same. The outer actuator beam is about 800 nm lower than the others (Figure 15a,b). This could be due to some remaining underneath the SiO${}_{2}$. We did notice some Si remaining underneath the SiO${}_{2}$ layer along the crossbar, due to differential etching rates. This could lead to the slightly curved crossbar (Figure 15c,d). Furthermore, the structures do have a gradient intrinsic stress. For very long structures, such as the crossbar, this might result in some curvature.

For a small-footprint configuration, such as the IBA with the 50 μm pitch, all suspended beams (with the same stacks and geometry) have the same free-end position. In addition, this configuration tends to have robuster manufacturing yield. For a large-footprint configuration with more suspended beams (e.g., the SBA configuration), the crossbar is not entirely flat. However, the WG beams that are located in the middle of the crossbar, and the relative post-release offset of their end-facets is still small. Hence, this configuration might still be useful for an alignment task, despite the deformation of the crossbar.

### 3.4. Motion of Separate Bimorph Actuator (SBA) and Integrated Bimorph Actuator (IBA)

The MEMS designs also allow rotational adjustment of the WG beam array along the optical axis by differentially powering the individual bimorph actuators. This is needed to compensate the chip pre-assembly error in that direction. This section compares the rotational adjustment of the SBA and IBA configuration, respectively.

In this experiment, a safe power was applied to drive the actuators to prevent any permanent change in the poly-Si resistance. The single power source we used provided a maximum total power of 120 mW was given to drive different number of actuators, and this power was distributed evenly to each activated pair of driven actuator beams. The vertical position of each of the WG end-facets was measured, by actuating a subset of the bimorph actuators. Each measurement point is the average result of five measurements on the free-end of the crossbar.

**Figure 16:**
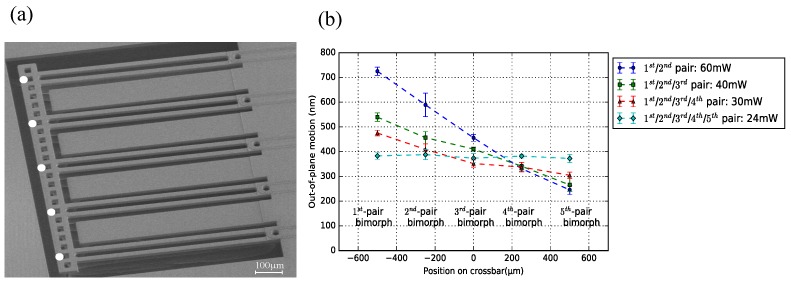
(**a**) SEM image of 5-pair DB bimorph actuators integrated with the WG beams. The measurement points on the crossbar are marked as the white dots; (**b**) crossbar shape indication with activating different actuator pairs.

A typical example of an IBA design ($W_{\mathrm{wgp}}$ = 250 μm, $L_{\mathrm{poly}}$ = 50 μm) is shown in Figure 16a, including 5-pair actuator beams (WG beams). Figure 16a depicts the measurement points observed with the vibrometer. Figure 16b shows the measurement results when actuating different combinations of actuator beams. Moreover, the standard deviation is included. As expected, by differential powering of the actuators, the angle of the cross-bar can be adjusted. Generally, with less pairs of actuator activated, the crossbar deforms more significantly. For example, using the chip body as reference plane, the crossbar has a rotational angle of 40° with 2-pair activated actuators, while the crossbar rotates 30° with 3-pair activated actuators. When all actuator pairs are driven with the same power, the crossbar keeps horizontally flat, confirming the outcome of former experiments. In this situation, the vertical offset among each WG end-facets are within ∼100 nm.

**Figure 17:**
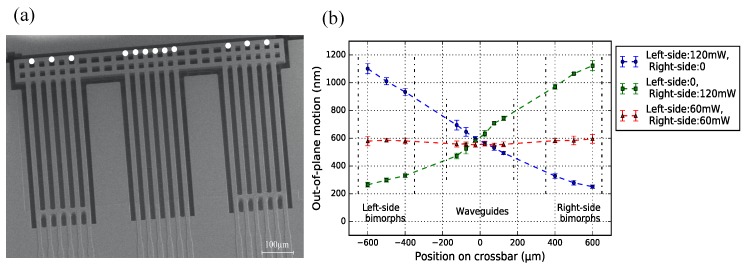
(**a**) SEM image of NDB bimorph actuators locating on both two sides of the WG array. The measurement points on the crossbar are marked as the white dots; (**b**) crossbar shape indication with activating different sets of actuators.

Figure 17a shows a SBA design ($W_{\mathrm{wgp}}$ = 50 μm, $L_{\mathrm{poly}}$ = 80 μm): three pairs of bimorph actuators are placed to each side of the WG array. Besides the end position of the WG beams, the end position of the bimorph actuators are also measured, to reflect the deformation of the entire crossbar. The measurements include either actuating both sets of actuators or only one set, and the results are shown in Figure 17b. When only one set of actuators are activated, the crossbar has the largest rotational angle, and this angle is measured to be the same (∼33°). The crossbar remains straight, particularly where the WGs are located.

## 4. Discussion

### 4.1. Motion Limit

The maximum motion range that can be achieved is determined by the maximum temperature the actuators can withstand. With an increased power input, more resistive heating is generated. The actuator will burn out when temperature reaches a certain point.

**Figure 18:**
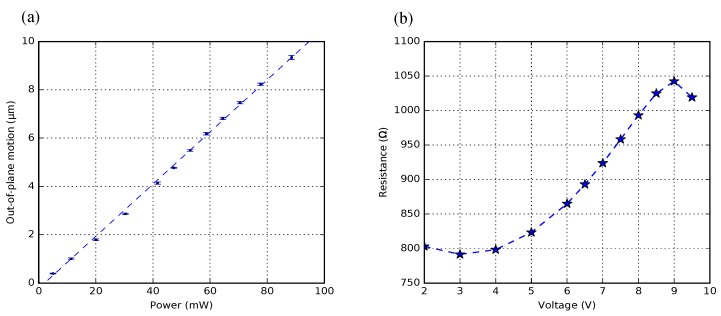
(**a**) motion characterization with standard deviation of a 1-pair bimorph actuator; (**b**) the measured electrical resistance of poly-Si (with 5 μm thickness) as a function of the actuator voltage.

**Figure 19:**
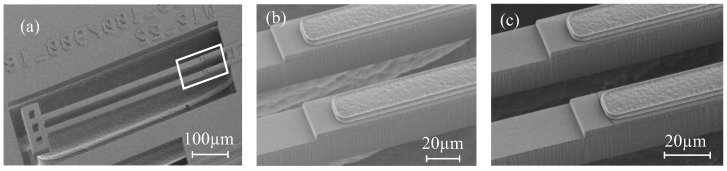
(**a**) SEM images of a 1-pair NBD bimorph actuator before any actuation; (**b**) close-up SEM image of the section where poly-Si ends, indicated in (**a**); (**c**) SEM image of the crack from (**b**), after a high power input.

To experimentally find the limit, a 1-pair BD-bimorph actuator (50 μm pitch, $L_{\mathrm{poly}}=90\;\mu m$) was measured, and its experimental results are presented in Figure 18. Each motion measurement point is the average result of five measurements on the free-end of the crossbar. The maximum deflection was measured to be 9.34 μm at 88.56 mW dissipated power. Figure 18a shows a linear relationship between the supplied power and the vertical deflection, and all the motion measurements have less than 50 nm standard deviation. The measured electrical resistance of poly-Si firstly shows a slight decrease and then a rapid raise, and eventually drops after reaching a peak (Figure 18b). During this experiment, the actuator started to emit visible light after applying 7 V. Further increase of the voltage increased light intensity until the actuator burnt out. This indicates that the poly-Si lattice is recrystallized when the temperature is elevated, which allows more excited electrons passing through. After the experiment, the actuator is electrically defective; mechanically, the overall actuator was still intact, although some cracks and delamination have occurred (Figure 19).

### 4.2. Bridge Design and Non-Bridge Design

To compare the performance of the BD and NBD, 1-pair bimorph actuator with the same poly-Si length ($L_{\mathrm{poly}}$ = 50 μm) were chosen. Before any resistive heating, the resistance of the NBD was measured to be 473 Ω, whereas the resistance of the BD was measured to be 655 Ω.

**Figure 20:**
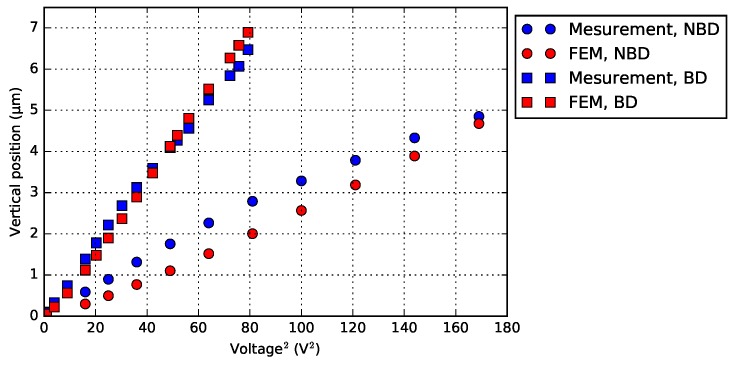
Motion characterization of a 1-pair bimorph actuator, with its FEM results.

**Figure 21:**
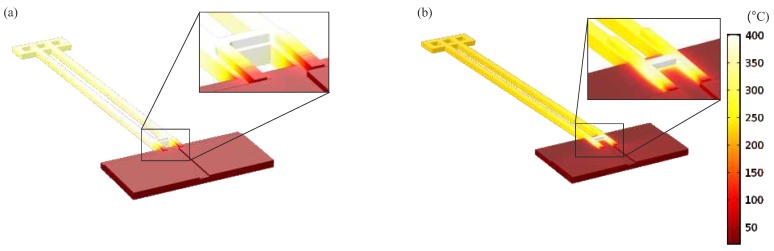
FEM temperature results of two variants based on the different heater locations: (**a**) DB configuration and (**b**) NDB configuration.

Only considering the vertical motion, the BD variant deflects more than the NBD, with the same voltage (Figure 20). The experimental results are verified by a FEM (finite element model) approach, using COMSOL Multiphysics^®^ (Version 5.2, COMSOL, Inc., Burlington, MA, USA). In all FEM, the heat flux and the heat transfer in materials are set under the same condition. Figure 21 shows the temperature gradients of both designs, when their heaters reach the same maximum temperature (i.e., 400 ${}^{\circ }C$). Since the heater is located on the base in the NBD variant, the actuator beams get heated less effectively than that of the BD.

### 4.3. Temperature Estimation

**Figure 22:**
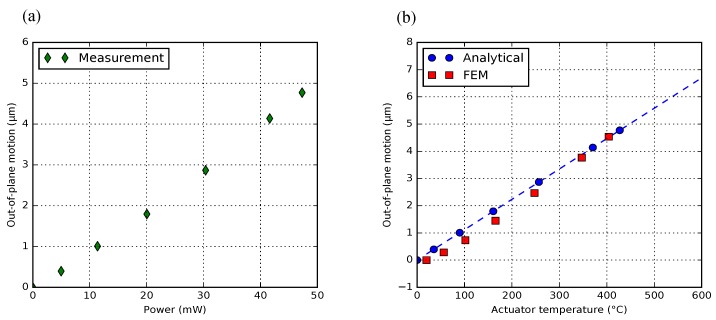
(**a**) motion characterization of a 1-pair BD bimorph actuator with a safe power supply; (**b**) the vertical deflection as a linear function of the analytically estimated actuator temperature. The FEM result is also presented.

To avoid the high resistive temperature that can destroy poly-Si lattices permanently, we prefer to operate the actuators safely below the thermal runaway temperature. To estimate the mean actuator temperature, the same model as the post-release deformation model was built (Appendix C). The modeling result shows that a linear deflection change of 10 nm/${}^{\circ }C$ for the poly-Si section with 5 μm in thickness and 60 μm in length. Figure 22a firstly shows the motion measurement of a 1-pair BD bimorph actuator within a safe voltage range, and by substituting the measured motion into the analytical model, Figure 22b shows the measured motion as a function of the analytically simulating actuator temperature, together with the FEM result. Both simulations show almost the same trend in the curve of motion and temperature.

### 4.4. Bimorph Actuator Pitch and Number of Pairs

**Figure 23:**
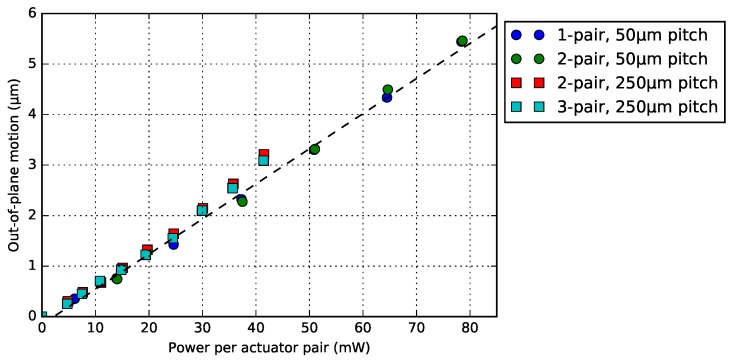
The consumed power of each pair of actuator as a function of the out-of-plane motion. The dashed line indicates a linear fit for the plots.

We have two WG-pitch system ($W_{\mathrm{wgp}}$) for our photonic application, and by activating different number of pairs of actuator beams ($N_{\mathrm{ba}}$), it is possible to fine-tune the WG end-position (Section 3.4). To compare the performance of the bimorph actuators with these two design variables, four BD bimorph actuators ($L_{\mathrm{poly}}$ = 60 μm) were characterized (Figure 23). For all experiments, each pair of bimorph actuator (with the same electrical resistance) was electrically connected in parallel. The comparison shows the motion as a function of power consumed by each pair of the actuator, and it corresponds with a linear fit. The results also show that each pair of the actuator generates almost the same vertical deflection when consuming the same power, regardless of the various $W_{\mathrm{wgp}}$ and $N_{\mathrm{ba}}$. As reported in Section 3.4, the design parameters ($W_{\mathrm{wgp}}$ and $N_{\mathrm{ba}}$) do affect the crossbar deformation during the actuation, though they do not influence the vertical motion.

### 4.5. Propagation and Coupling with Curved Beams

**Figure 24:**
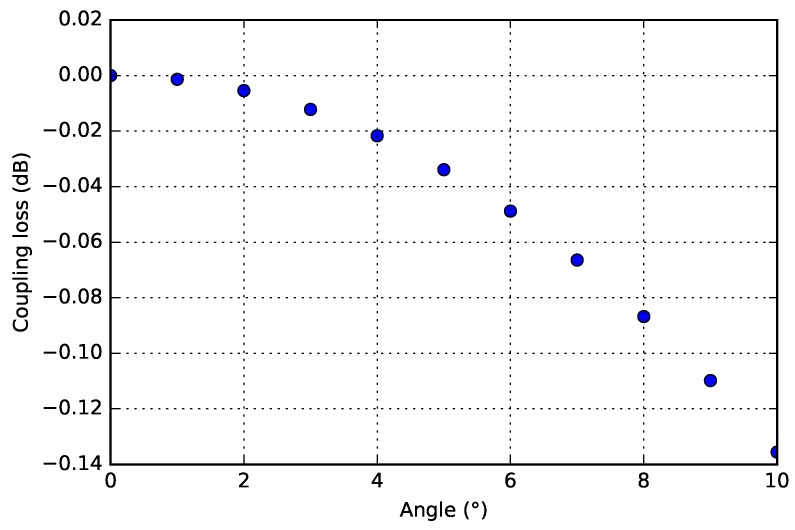
Modeling of coupling loss versus angular offset.

The curvature of the WG beams is small (∼230 /m and less) compared to the permissible curvature (up to ∼14,000 /m, depending on WG designs [2]) in the TriPleX material platform, and will not have any significant impact on the light propagation. The end-facets of the flexible WG beams will be under an angle with respect to the InP waveguide end-facets, for two reasons. Firstly, as part of the alignment process the flexible WG beams are bent, so the propagation direction is not perpendicular to the InP WG end-facet. However, this angle is very small and has an ignorable effect (e.g., 8 μm vertical deflection over a beam length of 900 μm, which corresponds to ∼0.5°). Secondly, the end-facet of the WG beams have a slope due to the etching process in which they are realized of ∼3.5°. For small mode fields, the sensitivity of coupling loss to angular error is more tolerant. Using the analytical models from [27], and taking a mode-field diameter of ∼1 μm at 1550 nm wavelength, shows that an angular offset of 5° leads to a coupling loss of less than 0.05 dB (Figure 24). Experimentally, our previous work has proven that the flexible WG beams and etched end-facets offer suitable mode-field diameters, and allow good optical coupling [10,28].

## 5. Conclusions

We have proposed a photonic alignment concept, which uses high-precision flip-chip bonding and on-chip MEMS actuation for pre-aligning the PICs and fine-positioning photonic WG beams, respectively. The out-of-plane and rotational actuation of the bimorph actuator is the focus of this paper. The design challenge of the bimorph actuator is to compensate the nominal offset between the WGs that need to be aligned, and the need of providing sufficient motion. We proposed an electrothermal actuator design, with short-loop poly-Si heaters.This concept is implemented in different designs, based on the location of the heaters and the integration of the actuators with the WG beams. The initial position of the WG end-facets and the motion range are both dependent on the same design parameter, $L_{\mathrm{poly}}$. For the specific application case, the results suggest that the optimal poly-Si length is approximately 30 μm, with a SiO${}_{2}$ length of 900 μm, for the PHASTFlex application. For other applications, obviously other values can be chosen.

Both IBA and SBA configurations have shown the capability of controlling the rotation of WG beams. With an evenly-assigned power on every actuator pair, the end-position of WG beams can maintain the same height. Having a more-compliant crossbar (e.g., with larger pitch in the IBA or the SBA), there can be more freedom in adjusting the WG rotation.

Moreover, an analytical single-beam model with multilayer stacks has been adjusted to estimate the post-release deformation. The model also suggests that, below 500 ${}^{\circ }C$–600 ${}^{\circ }C$ actuator temperature, the generated motion can be more than 5 μm, agreed well with the FEM. This can be beneficial for predicting the post-release deformation, as well as the actuation temperature for the application that is thermally governed.

## Figures and Tables

**Figure A1:**
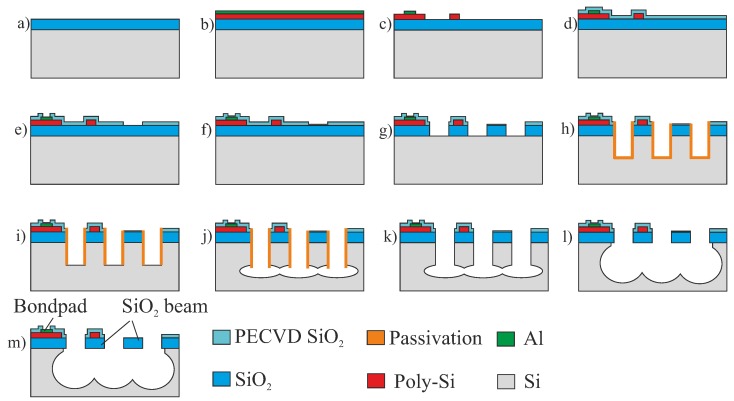
Main fabrication steps for realizing the SiO${}_{2}$-based MEMS. (**a**) Si wafer with 16 μm-thick SiO${}_{2}$; (**b**) deposition of poly-Si and Al; (**c**) patterning poly-Si and Al; (**d**) deposition of 2 μm-thick plasma-enhanced chemical vapor deposition (PECVD) SiO${}_{2}$; (**e**) local removal of PECVD SiO${}_{2}$; (**f**) deposition of ∼200 nm-thick PECVD SiO${}_{2}$; (**g**) patterning TriPleX SiO${}_{2}$; (**h**) anisotropic plasma etching of Si, and then deposition of passivation layer; (**i**) local removal of the passivation layer; (**j**) isotropic plasma etching of Si; (**k**) stripping off the passivation layer; (**l**) continuation of isotropic plasma etching of Si; (**m**) plasma etching of ∼200 nm-thick PECVD SiO${}_{2}$.

**Figure A2:**
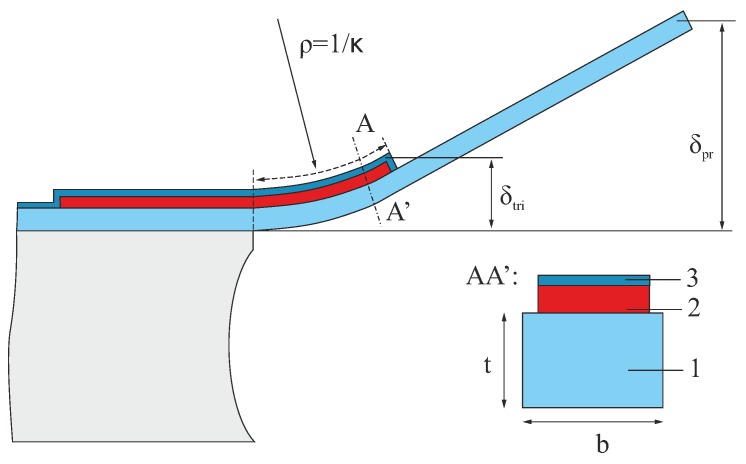
The illustration of the cross-section of the trilayer beam for analytical modeling.

## Abbreviations

The following abbreviations are used in this manuscript:

PIC	Photonic integrated circuit
MEMS	Micro-electromechanical systems
LTCC	Low Temperature Co-fired Ceramics
RF	Radio frequency
I/O	Input/Output
SEM	Scanning electron microscope
PECVD	Plasma-enhanced chemical vapor deposition
CTE	Coefficient of thermal expansion
WG	Waveguide
BD	Bridge design
NBD	Non-bridge design
IBA	Integrated bimorph actuator
SBA	Separate bimorph actuator

## Appendix A. Main Design Parameters

The dimension of the main design parameters are determined by photonic and mechanical demands, as summarized in Table A1.

**Table A1:** Overview of the main designed parameters and values of the waveguide (WG) beams and bimorph actuators. The values are chosen based on photonic (p) and/or mechanical (m) demands.

Parameter	Symbol	Value	Rationale
Number of WG beams	$N_{\mathrm{wg}}$	2/4/6	p + m
WG beam thickness (μm)	$t_{\mathrm{ox}}$	16	p
WG beam width (μm)	$W_{\mathrm{ox}}$	22	p + m
WG beam length, including crossbar (μm)	$L_{\mathrm{wg}}$	900	m
Crossbar width (μm)	$W_{t}$	72	m
WG beam pitch (μm)	$W_{\mathrm{wgp}}$	50/250	p
Number of actuator pairs (two beams per pair)	$N_{\mathrm{ba}}$	1/2/3	m
Poly-Si width	$W_{\mathrm{poly}}$	12	m
Poly-Si thickness (μm)	$t_{\mathrm{poly}}$	5	m
Poly-Si length (μm)	$L_{\mathrm{poly}}$	20–200	m
Actuator beam pitch (μm)	$W_{\mathrm{bap}}$	50	m
Distance between WG and actuators (μm)	$W_{\mathrm{wgba}}$	250	p + m

## Appendix B. Fabrication Process

Figure A1 illustrates the main steps of the MEMS fabrication on the front-side of the TriPleX wafer. The 16 μm-thick SiO${}_{2}$ is actually made of a layer of 8 μm-thick thermally-grown SiO${}_{2}$ as a bottom cladding on a Si substrate, and another 8 μm-thick PECVD SiO${}_{2}$ as a top cladding. These two SiO${}_{2}$ stacks are annealed, to obtain uniform optical and mechanical properties (Figure A1a). The structures illustrated in Figure A1 represent a bondpad, a SiO${}_{2}$ beam with poly-Si and a SiO${}_{2}$ beam without poly-Si.

On the front-side of the TriPleX wafer, 5 μm-thick poly-Si is firstly deposited, followed by the deposition of Al with 1 μm thickness (Figure A1b). The Al stack is used for building electrical leads. After this, Al and poly-Si are patterned (Figure A1c). As mentioned in Section 2.2, to protect the poly-Si layer during the subsequent Si etching steps to release the suspended structures, a layer of 2 μm PECVD SiO${}_{2}$ is used to cover the entire wafer (Figure A1d), and then the areas where the bondpad and suspended structure without the poly-Si pattern are located are opened by plasma etching (Figure A1e). Removing the PECVD SiO${}_{2}$ layer on the bondpad enables the electrical probing and wire-bonding with PCBs (Printed Circuit Boards) [9]. As the PECVD SiO${}_{2}$ stack causes additional post-release deflection, for the SiO${}_{2}$-only section without poly-Si, there is no need to keep this PECVD SiO${}_{2}$. This is also a major difference, between the front-side recipe that was used before [8,9,10] and the current one that needs to facilitate the post-release deformation within the PHASTFlex alignment scheme.

After this local removal of SiO${}_{2}$, another very thin SiO${}_{2}$ (∼200 nm) is deposited (Figure A1f). Figure A1g shows how the SiO${}_{2}$ stack is patterned. The Si-reinforced release method is depicted from Steps h to j (Figure A1), by introducing a passivation layer to reinforce the SiO${}_{2}$-Si stack when it is released from the Si substrate. Once the suspended structures are released, the passivation is removed (Figure A1k), and the remaining Si underneath the SiO${}_{2}$ stack is isotropically etched away completely (Figure A1l). Lastly, the bondpad and beam structure without the Si pattern are opened again (Figure A1m). A slight over-etch is proceeded to expose the bondpad.

## Appendix C. Analytical Multilayer Single-Beam Model

The curvature (κ) and the radius of curvature (ρ) of a single beam made of multiple stacks can be described by [23,24]:

(A1) $$\kappa =\frac{1}{\rho }=\frac{2RA^{-1}S}{2+RA^{-1}B},$$

with

(A2) $$R=\left[\begin{array}{c} \frac{t_{1}}{2}\\ t_{1}+\frac{t_{2}}{2}\\ t_{1}+t_{2}+\frac{t_{3}}{2} \end{array}\right]\;\frac{-1}{E_{1}I_{1}+E_{2}I_{2}+E_{3}I_{3}},$$

(A3) $$A=\left[\begin{array}{ccc} {\left(E_{1}I_{1}\right)}^{-1} & -{\left(E_{2}I_{2}\right)}^{-1} & 0\\ 0 & {\left(E_{2}I_{2}\right)}^{-1} & -{\left(E_{3}I_{3}\right)}^{-1}\\ 1 & 1 & 1 \end{array}\right]\;,$$

(A4) $$S=\left[\begin{array}{c} \epsilon_{2}-\epsilon_{1}\\ \epsilon_{3}-\epsilon_{2}\\ 0 \end{array}\right]\;,$$

(A5) $$B=\left[\begin{array}{c} t_{2}+t_{1}\\ t_{3}+t_{2}\\ 0 \end{array}\right]\;,$$

(A6) $$I_{i}=\frac{b_{i}t_{i}^{3}}{12},$$

(A7) $$A_{i}=b_{i}t_{i},$$

(A8) $$\epsilon_{i}=\alpha_{i}\Delta T\;\left(\mathrm{for}\;\mathrm{thermal}\;\mathrm{effect}\right),$$

(A9) $$\epsilon_{i}=-\frac{\sigma_{i}}{E_{i}}\;\left(\mathrm{for}\;\mathrm{residual}\;\mathrm{stress}\right),$$

where $E_{i}$, $I_{i}$, $A_{i}$, $b_{i}$, $t_{i}$ and $\alpha_{i}$ are the Young’s modulus, moment of inertia, cross-section area, width, thickness and coefficient of thermal expansion (CTE) of layer *i*, respectively. Moreover, $\epsilon_{i}$ is strain of material *i*, mainly resulting from residual stress and thermal effects. ΔT is the temperature cycles that the beam undergoes during fabrication or actuation. Figure A2 describes the simplified cross section of the trilayer beam, and Table A2 includes the measured dimensions and material properties of the multi-stack beam. The end-deflection ($\delta_{\mathrm{tri}}$) of the trilayer stacks (TriPleX SiO${}_{2}$/poly-Si/PECVD SiO${}_{2}$) can be approximated by:

(A10) $$\delta_{\mathrm{tri}}=\rho \left(1-\cos\left(\frac{L_{\mathrm{poly}}}{\rho }\right)\right),$$

where $L_{\mathrm{poly}}$ is the length of the poly-Si section (Table A1). As mentioned in Section 2.3, as the rest of the beam only includes SiO${}_{2}$, it can be simplified as a straight beam. Thus, the total deflection ($\delta_{\mathrm{pr}}$) of the beam can be concluded by

(A11) $$\delta_{\mathrm{pr}}=\delta_{\mathrm{tri}}+\left(L_{\mathrm{wg}}-L_{\mathrm{poly}}\right)\sin\left(\frac{L_{\mathrm{poly}}}{\rho }\right),$$

where $L_{\mathrm{wg}}$ is the length of the entire beam (Table A1).

**Table A2:** Measured dimensions and material properties of the multilayer beam.

Layer	1	2	3
Material	TriPleX (SiO${}_{2}$ )	Poly-Si	PECVD SiO${}_{2}$
Beam width: *b* (μm)	∼20 μm	∼12 μm	∼12 μm
Beam thickness: *t* (μm)	16	5	2
Young’s Modulus: *E* (GPa)	70	160	75
Coefficient of thermal expansion (CTE): α ($10^{-6}$ /${}^{\circ }C$)	0.5	3.44	2.5
Residual stress *: σ (MPa)	−19.2	56.1	61.5

* The residual stress is approximated by the measured/simulated mean intrinsic stress.
